# Tactile Motion and Pattern Processing Assessed with High-Field fMRI

**DOI:** 10.1371/journal.pone.0024860

**Published:** 2011-09-15

**Authors:** Evelin Wacker, Bernhard Spitzer, Ralf Lützkendorf, Johannes Bernarding, Felix Blankenburg

**Affiliations:** 1 Department of Neurology and Bernstein Center for Computational Neuroscience, Charité, Berlin, Germany; 2 Institute for Biometry and Medical Informatics, Otto-von-Guericke University, Magdeburg, Germany; Queensland Institute of Medical Research, Australia

## Abstract

Processing of motion and pattern has been extensively studied in the visual domain, but much less in the somatosensory system. Here, we used ultra-high-field functional magnetic resonance imaging (fMRI) at 7 Tesla to investigate the neuronal correlates of tactile motion and pattern processing in humans under tightly controlled stimulation conditions. Different types of dynamic stimuli created the sensation of moving or stationary bar patterns during passive touch. Activity in somatosensory cortex was increased during both motion and pattern processing and modulated by motion directionality in primary and secondary somatosensory cortices (SI and SII) as well as by pattern orientation in the anterior intraparietal sulcus. Furthermore, tactile motion and pattern processing induced activity in the middle temporal cortex (hMT+/V5) and in the inferior parietal cortex (IPC), involving parts of the supramarginal und angular gyri. These responses covaried with subjects' individual perceptual performance, suggesting that hMT+/V5 and IPC contribute to conscious perception of specific tactile stimulus features. In addition, an analysis of effective connectivity using psychophysiological interactions (PPI) revealed increased functional coupling between SI and hMT+/V5 during motion processing, as well as between SI and IPC during pattern processing. This connectivity pattern provides evidence for the direct engagement of these specialized cortical areas in tactile processing during somesthesis.

## Introduction

Human somatosensation can supply the organism with information about “where” (e.g., on the left forearm), “what” (e.g., a raindrop or an insect), and “how” (e.g., moving towards the hand) environmental stimuli are experienced. Compared to vision, however, the neuronal pathways underlying the processing of specific tactile stimulus attributes are still largely controversial. The best studied dimension of somatosensory perception is the location of tactile stimuli on the body surface, which is long known to be represented in a somatotopic manner (“sensory homunculus”) in the postcentral gyrus of the primary somatosensory cortex (SI) [1] and in the parietal operculum of the secondary somatosensory cortex (SII) [2]. SI comprises multiple contralateral body representations [3]–[6] in four cytoarchitectonically different areas (Brodmann areas 3a, 3b, 1, and 2 [7]) with different functional roles and a postulated hierarchy, according to which area 3b can be regarded as “SI proper” [8]. Similarly, somatotopic representations were also found in several subdivisions of SII (e.g., parietal ventral area and area S2 [9]), which were more recently cytoarchitectonically characterized in humans as OP 1, OP 2, and OP 4 [10].

However, besides the neuronal representation of tactile location, there is accumulating evidence that also aspects of the remaining stimulus dimensions, such as motion and pattern, are coded already in SI and SII. For instance, neurophysiological studies in monkeys have identified populations of SI neurons whose responses are modulated by the direction of stimulus motion [11]–[13]. More recently, orientation-tuned neurons have been found in SI [14], [15] and SII [16], [17], and SI has been shown to play an important role in tactile pattern recognition [18]–[20]. Human neuroimaging studies have consistently demonstrated activity in SI and SII related to the discrimination of moving tactile stimuli [21], [22], and SI has been associated with the processing of tactile form [23], [24]. The involvement of SII in tactile pattern discrimination is however not yet fully elucidated; there is evidence both for [24] and against it [25].

Apart from SI and SII, the course of tactile motion and pattern processing is less clear. There is some evidence that the anterior part of the supramarginal gyrus in the inferior parietal cortex (IPC) is involved in tactile discrimination of shapes and/or form [23], [24], [26] and lesion studies indicated that there are somatosensory association areas in the IPC assumed to be specific to tactile shape processing [27], [28].

In addition, functional magnetic resonance imaging (fMRI) studies have shown that the processing of tactile stimulus features is often characterized by activity in areas that are traditionally associated with visual equivalents of these features. Tactile motion, for example, has been found to engage area hMT+/V5 in the middle temporal cortex, both in sighted [29]–[31] and congenitally blind individuals [32]–[34]. First identified as responsive to visual motion in the middle temporal cortex of the monkey [35], area MT/V5 and neighboring motion-sensitive areas such as the medial superior temporal area (MST) were collectively termed MT+/V5. The human homologue of this region, identified using (non-invasive) neuroimaging in humans [36]–[38] has long been considered a purely visual motion-sensitive area. Similarly, processing of tactile shapes typically activates extrastriate areas [39]–[42] such as the lateral occipital complex (LOC), which plays a crucial role in visual object shape perception. One possible explanation for the recruitment of these specialized visual areas during processing of tactile stimulation may be visual imagery of tactile stimulus features (e.g., [43]; but see [44]), but the actual function of these areas during tactile information processing remains poorly understood.

Recent findings suggest that somatosensory processing of tactile motion and pattern may also involve areas that are not directly associated with any specific sensory modality. One such multisensory region is the intraparietal area lining the intraparietal sulcus (IPS) in the posterior parietal cortex. The IPS contains multiple parietal fields, among others the anterior and the ventral intraparietal areas (AIP and VIP), which are better characterized in monkeys than in humans [45]–[47]. However, Bremmer and colleagues identified a ventral area in the human anterior IPS (aIPS) involved in visual, tactile, and auditory motion processing [48] that might be equivalent to the motion-sensitive multisensory association area VIP in monkeys. Likewise, similar to the monkey AIP, which is highly responsive to size, shape, and orientation of objects, parts of the human aIPS were shown to be engaged in visual and tactile discrimination of grating orientation [49], [50], in form perception [23], [24], and in object recognition [51], [52].

Despite these recent advances in delineating the cerebral networks engaged in tactile motion and pattern processing, a clear consensus regarding the specific processing pathways is still lacking. This may in particular be due to large methodological differences regarding stimulus characteristics (two- or three-dimensional stimuli), task (discrimination, recognition, or naming), and exploratory strategies (passive or active, single digit or whole hand). In fact, compared to studies of vision, experimental investigation of tactile sensations is often complicated by the problem of mechanically administering well-described and replicable cutaneous input that creates the percept of interest (such as motion, shape, or object orientation), ideally unconfounded by active motor exploration.

Here, we investigated the processing of tactile motion and pattern using a fingertip-sized multi-pin stimulation device similar to a Braille display to induce the sensation of moving and stationary bar patterns within a circumscribed area of glabrous skin. The percepts of interest were created during passive touch under fully specified physical stimulus conditions, thereby achieving a high level of control over the mechanical input. Using a passive stimulation paradigm, not requiring any overt response to the stimuli of interest, further ensured that the results were not affected by response-induced BOLD signal changes in the somatosensory system. Both for moving and for stationary patterns, matched control stimuli were designed that preserved the overall physical dynamics of the stimuli of interest but did not induce a percept of motion or pattern (see Materials and Methods), allowing us to contrast motion- and pattern-specific activity in a balanced experimental design.

For a fine-grained analysis of the neuronal networks engaged in processing of tactile motion and patterns, we utilized ultra-high-field fMRI at 7 Tesla. In the analysis, we investigated to what extent specialized visual and/or multisensory areas are recruited under tightly controlled tactile stimulation conditions, and sought to determine the relations between subject-specific brain activity in these areas and the outcome of individual behavioral performance in identifying the stimulus attributes of interest. Furthermore, we studied stimulus-induced changes in effective connectivity between these specialized cortical areas and somatosensory cortices in order to characterize their functional integration in tactile information processing.

## Materials and Methods

### Participants

Thirteen healthy volunteers (aged between 22 and 35 years; nine males, one left-handed) participated in the study with written informed consent. The study corresponded to the Human Subjects Guidelines of the Declaration of Helsinki and was approved by the Local Ethics Committee at the faculty of medicine, Otto-von-Guericke-University of Magdeburg.

### Stimuli

**Figure 1:**
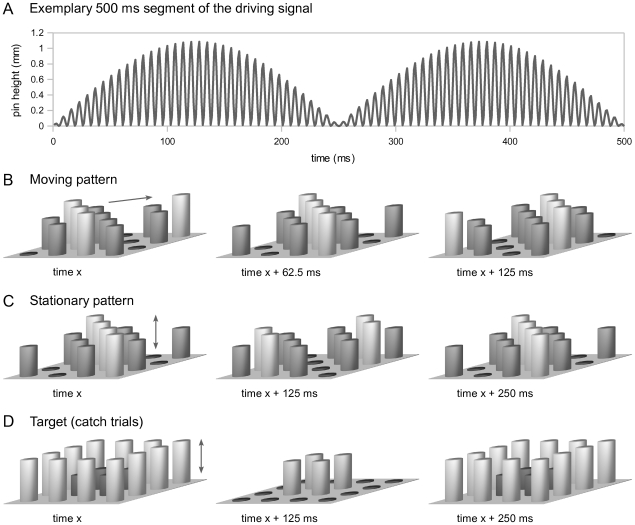
Illustration of tactile stimuli used. A. The pins' driving signal was a 144 Hz sinusoidal carrier, which was amplitude-modulated by a rectified 2 Hz sine function. B. A directed propagation of the diagonals' sine phase across the display plane resulted in a percept of a bar pattern travelling smoothly across the fingertip. Both upward and downward diagonal orientations were used corresponding to orthogonal moving directions. For moving random stimuli, each of the four sine phases was assigned to four randomly chosen pins (not shown). C. Diagonals oscillating at opposite phases created the percept of a stationary bar pattern, which was periodically elevated and retracted. Again, both diagonal orientations were used. For stationary random stimuli, the driving signals were assigned to sets of randomly chosen pins (not shown). D. The target stimulus (to be detected on infrequently presented “catch” trials) was an oscillating square.

Tactile stimulation was applied to the left index finger by a 16-dot piezoelectric Braille-like display (4×4 quadratic matrix, 2.5 mm spacing) controlled by a programmable stimulation device (Piezostimulator, QuaeroSys, St. Johann, Germany). On each trial, the pins of the display were driven for 4000 ms by a 144 Hz sinusoidal carrier signal, which was amplitude-modulated by different sets of rectified 2 Hz sine functions (see Figure 1A for illustration). Four different stimulus types were designed to create the sensation of (1) a moving bar pattern, (2) a moving random stimulus, (3) a stationary bar pattern, or (4) a stationary random stimulus.

For the moving bar pattern (1), all pins forming a diagonal on the quadratic display were driven by a rectified 2 Hz sine function, with half cycles repeating every 250 ms. From diagonal to diagonal, the phase of the sine function was shifted by π/4 (62.5 ms, corresponding to a quarter of its rectified half cycle; see Figure 1B for illustration). This directed propagation of the diagonals' across the display plane created the sensation of a bar pattern travelling smoothly across the fingertip. The orientation of the diagonals and the direction of movement were randomly varied from trial to trial. For the moving random stimulus (2), the same set of driving signals was used as for (1), but each of the four different sine phases was randomly assigned to four randomly chosen pins. This corresponded to a random spatial permutation of the individual pin movements displayed in (1) and created a percept of unsystematic, disorderly motion across the display plane.

For the stationary bar pattern (3), every second diagonal of the display was driven identically to (1), thus oscillating at opposite phases of the half cycle. The remaining pins, however, (i.e., the interleaved diagonals) were all synchronously driven by the same signal (Figure 1C). As a result, due to the absence of a directed phase shift, the bar pattern did not propagate across the display plane but created the percept of a stationary pattern, which was periodically elevated and retracted (i.e., along the z-axis). In order to ensure identical root mean square (RMS) amplitudes in each stimulus condition at each time point, the instantaneous amplitude of the interleaved diagonals in (3) was set to the average of the corresponding driving signals used in (1). For the stationary random stimulus (4), the driving signals used for (3) were randomly assigned to randomly chosen pins, creating a percept of a disorderly structured surface, which was periodically elevated and retracted. The four stimulus types were matched according to the overall physical dynamics rather than to the subjectively perceived salience of the different stimulus attributes in order to allow for a priori unbiased investigation of covariations between the fMRI results and subjects' individual perceptual performance.

During scanning, presentation of the four stimulus types was not associated with any behavioral task. To ensure that participants maintained attention to the tactile stimulation throughout the scanning sessions, they were instructed to report the occurrence of a distinct tactile stimulus, which was infrequently presented (“catch trials”); see Figure 1D for illustration. The to-be-detected target shape was markedly different from the stationary or moving gratings of interest. It consisted of a square, with the outer pins of the display oscillating at 2 Hz (rectified), while the inner pins of the display were driven such that across the stimulation period, the average RMS amplitude of the target stimulus was identical to the average RMS amplitude of the four main stimuli. Stimulus presentation was controlled using custom MATLAB code (The MathWorks) and the Cogent 2000 toolbox (http://www.vislab.ucl.ac.uk/cogent.php).

### Behavioral data

Prior to the experiment, participants were familiarized with the stimulation device and with the different types of stimuli. Before and after scanning, subjective discriminability of the stimuli was assessed using a behavioral classification task (pre- and post-test; 48 trials each). To avoid ceiling effects, the four main stimulus types were presented in a four-alternative classification task, which was considerably more difficult than that of detecting the presence/absence of the two stimulus features of interest (motion and/or pattern). Individual perceptual performance levels for motion and pattern identification were inferred from the four-alternative classification data by collapsing correct classifications of the stimulus attribute of interest while disregarding false classifications along the other stimulus dimension.

### Design & Procedure

The fMRI experiment consisted of three sessions. Each session comprised 64 stimuli (16 of each stimulus type: moving bar patterns, stationary bar patterns, moving random stimuli, and stationary random stimuli), 16 null events, as well as 4 catch trials, in which the to-be-detected target shape was presented. All trials, including null events, had a duration of 4000 ms and were presented in pseudo-random serial order, such that each type of event occurred equally often in each quarter of the session. The inter-stimulus interval was randomly varied between 2500 and 7500 ms. Participants were instructed to keep their eyes closed throughout the experiment and to press a response key with their right index finger only when they detected the target stimulus, which was presented infrequently.

### fMRI data acquisition

Functional imaging was performed on a 7 Tesla Magnetom MRI scanner (Siemens, Erlangen, Germany) with a 24-channel head-coil system (Nova Medical, Wilmington, MA, USA). T2*-weighted functional images were acquired using an echo planar imaging (EPI) sequence (TR = 2500 ms, TE = 21 ms, flip angle  = 80°). For each session, 340 EPI volumes were obtained, each consisting of 46 axial slices covering the whole brain in an ascending order (slice thickness 2.5 mm, distance factor 0.25, in-plane resolution 2×2 mm, matrix size 106×106). To achieve this high spatial resolution with single-shot EPI acquisition, parallel imaging (GRAPPA) with an acceleration factor of two and a partial Fourier acquisition scheme (75%) were applied.

### Data analysis

Functional images were preprocessed and analyzed using Statistical Parametric Mapping (SPM8, Wellcome Department of Imaging Neuroscience, University College London, UK). The first four volumes of each experimental session were discarded in order to allow the T1-relaxation to reach equilibrium. To minimize movement-induced image distortions, each data set was realigned to the first image of the first session using a least-squares approach and a 6-parameter (rigid body) spatial transformation. The realigned images were spatially normalized to the standard Montreal Neurological Institute (MNI) template brain and smoothed using an isotropic, three-dimensional Gaussian kernel of 2 mm full-width at half-maximum (FWHM). This small kernel size was chosen to do not blur the fine-grained spatial resolution of the 7 T data. To remove global effects from the fMRI time series, detrending was applied based on a voxel-level linear model of global signal (LMGS) [53]. The images were high-pass filtered (cut-off frequency 128 s) in order to remove low-frequency signal drifts. To reduce high-frequency noise, serial correlations were modeled using an autoregressive AR(1) model.

A standard two-level mixed-effects model [54] was used for statistical analysis. At the first level, multiple regression within the framework of the general linear model (GLM) was employed to implement a within-subject analysis. For each data set, BOLD responses were modeled by stick functions (multiplied by the stimulus duration) indicating the onsets of the stimuli of interest (i.e., moving bar patterns, moving random stimuli, stationary bar patterns, stationary random stimuli). These regressors were then convolved with a standard hemodynamic response function (HRF) and included in the GLM. Two further regressors were added as modulatory effects, indicating the orientation (upward or downward diagonal) of moving and stationary patterns. Null events were explicitly modeled with a separate regressor to obtain a baseline. A stimulus function for nuisance effects comprised catch trials and accidental button presses. To account for occasional signal intensity changes within slices due to increased susceptibility-induced frequency variations at higher field strengths [55], seven additional nuisance regressors were included. These corresponded to the first seven eigenvariates, which were extracted exclusively from signals outside the brain in a previous SPM analysis (thresholded at p<0.05). After the model was fitted to the experimental data, contrast images were generated from the stimulus functions' parameter estimates for each of the four stimulus types of interest. At the second level, the individual subjects' contrast images were entered into a 2×2 within-subjects ANOVA with factors *motion* (moving/stationary) and *pattern* (patterned/random). This allowed computing differential effects between the different stimulus types, using contrast vectors to produce Statistical Parametric Maps (SPMs). An additional covariate comprised the individual subjects' accuracy levels for the classification of the four stimulus types (mean of pre- and post-test) to account for variance due to performance differences.

To identify the overall neuronal network involved in somatosensory perception, contrast images were generated at the subject level in order to compare the tactile stimulation conditions with the null events. At the group level, a one-sample t-test was calculated using the individual subjects' contrast images.

Effect sizes within activated clusters were calculated as percent signal change using the rfxplot toolbox [56] for SPM8. The same toolbox was used to extract BOLD time courses. The data were averaged across subjects and plotted for voxels showing the individual activation maximum within a sphere of 5 mm radius constructed around the group-level activation maximum. The mean-corrected BOLD time courses were plotted time-locked to tactile stimulation onset (stimulus duration was 4 s). The errors plotted as dotted lines around the mean response correspond to +/- 1 standard error of the mean.

To assess possible BOLD differences for motion direction and pattern orientation, the individual subjects' contrast images that were generated from the two regressors indicating bar orientation for moving patterns and stationary patterns were entered into another ANOVA. To consider effects of both directions/orientations F-contrasts were computed. These contrasts were examined within the activation maps that resulted from contrasting moving with stationary stimuli and patterned with random stimuli to identify differential effects within motion- and pattern-specific areas.

To investigate performance-dependent covariation in regions involved in tactile motion and pattern processing, the individual subjects' accuracy in identifying moving and patterned stimuli correctly outside the scanner (inferred from the mean classification performance in the behavioral pre- and post-test) was correlated with the individual subjects' parameter estimates of peak voxels in the areas identified for motion and pattern processing, respectively (hMT+/V5; x = −44, y = −70, z = −2 and IPC; x = −60, y = −56, z = 32). These tests were Bonferroni corrected for multiple comparisons.

The effective connectivity of areas involved in motion and pattern processing was assessed using psychophysiological interaction (PPI) analyses [57]. Spheres with a radius of 5 mm constructed around peak voxels of the motion and pattern activation located in left hMT+/V5 (x = −44, y = −70, z = −2) and left IPC (x = −60, y = −56, z = 32) served as seed regions for extracting the first eigenvariate of the signal. At the subject level, the physiological variable was extracted and psychophysiological interaction terms were created for moving vs. stationary stimuli as well as for patterned vs. random stimuli. Subsequently, these terms were entered into GLMs. At the group level, contrast images of the PPIs of the individual subjects were analyzed using one-sample t-tests.

All reported coordinates correspond to the anatomical MNI space. The SPM anatomy toolbox [58] was used to establish cytoarchitectonic reference where possible. To investigate the overall effects of tactile stimulation, we used a significance threshold of p_cluster_<0.05, family-wise error (FWE) corrected. To investigate stimulus-specific differences within the network associated with tactile stimulation, this contrast was used as a mask and, based on a priori assumptions (e.g., [22], [23]), the significance threshold was chosen more liberally (p<0.005, uncorr.). This threshold was also used for the additional analysis of BOLD signal changes for motion direction and pattern orientation. To assess differential effects between stimulus conditions regarding the entire brain, that is, also including areas that may not have been generally activated by tactile stimulation, we used solely the conservative threshold of p_cluster_<0.05, whole-brain FWE corrected. This threshold was also used for the PPI analyses.

## Results

### Behavioral data

On average, perceptual performance (inferred from the mean classification performance before and after scanning) was 71% (SEM = ±3%) in discriminating moving from stationary stimuli and 72% (SEM = ±3%) in discriminating patterned from random stimuli. No significant differences between the performance levels in the pre- and post tests were observed (p = 0.43 and p = 0.18).

Regarding the target detection task during the fMRI experiment, participants detected on average 77% of the 4 catch trials per session correctly. Accidental button presses during the presentation of the main stimuli were rare (2.5%).

### fMRI data

**Figure 2:**
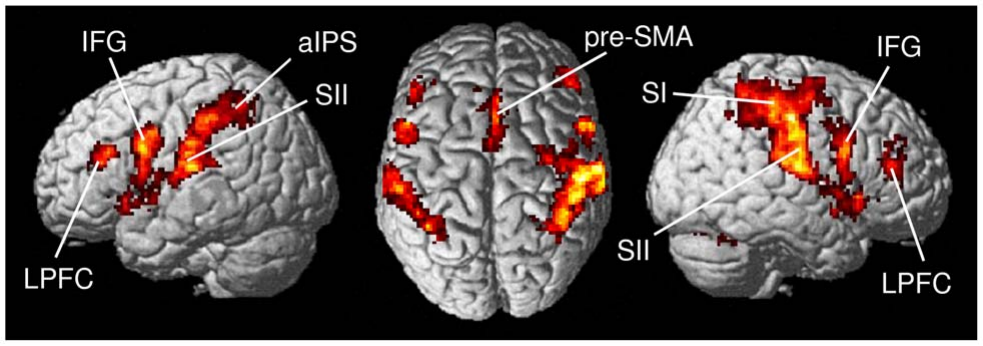
Overall neuronal network associated with tactile stimulation. Contrasting tactile stimulation trials with null events revealed a distributed network involved in tactile information processing, including contralateral SI and bilateral SII, anterior intraparietal sulcus (aIPS), inferior frontal gyrus (IFG), lateral prefrontal cortex (LPFC), pre-supplementary motor area (pre-SMA), insular cortex, thalamus, and cerebellum. (Group-level analysis; p_cluster_<0.05, whole-brain FWE corr.).

To identify the overall neuronal network involved in somatosensory perception, all tactile stimulation conditions were contrasted with the null events. In line with previous findings [24], [44], [59], this contrast revealed increased activation in contralateral primary somatosensory cortex in postcentral gyrus (SI; areas 3b, 1, 2) and in bilateral secondary somatosensory cortex/parietal operculum (SII; OP 1, 4), as well as in anterior intraparietal sulcus (aIPS; areas hIP3, hIP2), inferior frontal gyrus (IFG; area 44), lateral prefrontal cortex (LPFC), pre-supplementary motor area (pre-SMA; area 6), insular cortex, thalamus, and cerebellum in both hemispheres (see Figure 2 and Table 1).

**Table 1:** Functional regions active during tactile stimulation.

Region	Hemisphere	x	y	z	T-value
Primary somatosensory cortex	R	52	−22	44	12.61
Secondary somatosensory cortex	R	52	−16	16	10.29
	L	−58	−20	16	10.30
Anterior intraparietal sulcus	R	40	−48	62	8.85
	L	−38	−50	48	6.55
Inferior frontal gyrus	R	56	10	22	9.79
	L	−52	12	30	11.63
Lateral prefrontal cortex	R	42	40	16	5.81
	L	−42	32	18	7.59
Pre-supplementary motor area	R/L	2	16	46	7.56
Insular cortex	R	36	22	−2	9.25
	L	−36	18	−2	6.02
Thalamus	R	10	−14	4	5.81
	L	−10	−12	−2	5.38
Cerebellum	R	24	−66	−24	8.53
	L	−24	−50	−28	9.95

x, y, z are MNI coordinates (mm). T-values are local maxima within a significant cluster of activated voxels with p_cluster_<0.05, FWE corr. (group-level analysis). R = right hemisphere, L = left hemisphere.

**Figure 3:**
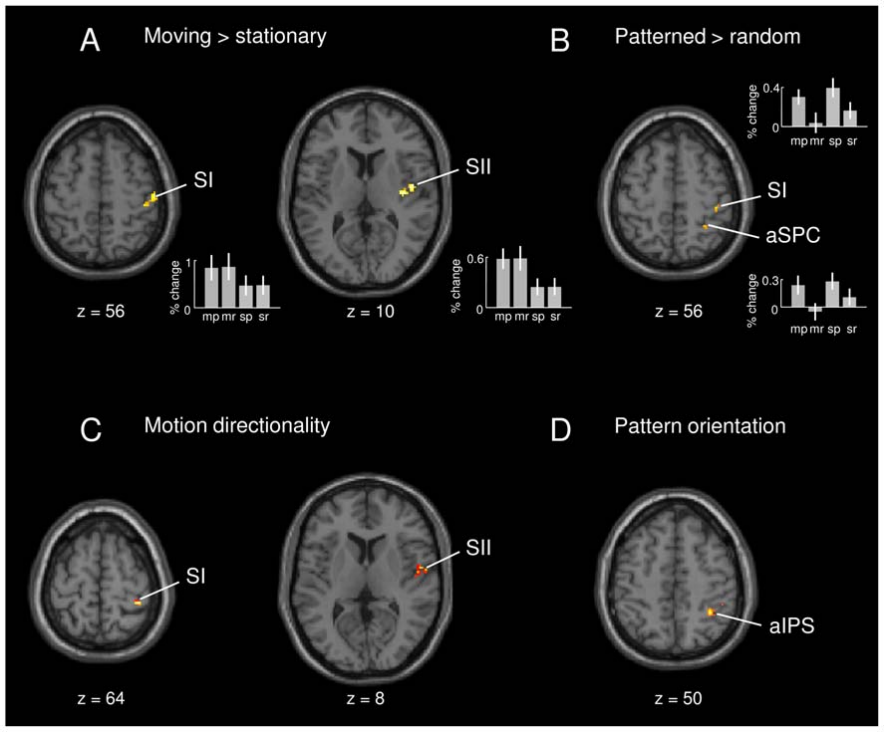
Motion- and pattern-specific differences and differential effects for motion direction and pattern orientation. A–B. Differential effects within the network associated with tactile stimulation (shown in Figure 2). Contrasting moving with stationary trials revealed an increased BOLD response in contralateral SI and SII (A). Contrasting patterned with random stimulus trials showed an increased BOLD response in anterior superior parietal cortex (aSPC) and SI (B). Effect sizes are plotted in terms of % signal change for all four stimulus types: moving patterned (mp), moving random (mr), stationary patterned (sp), and stationary random (sr). C–D. Differential effects for motion direction and pattern orientation. The regressor for moving pattern orientation revealed directionality effects for moving patterns in SI and SII (C). Differential effects for pattern orientation of stationary patterns were found in anterior intraparietal sulcus (aIPS; D). (Group-level analysis; p<0.005, uncorr.).

Assessing stimulus-specific differences within the network identified above, the comparison between moving and stationary stimuli revealed an increased BOLD response in contralateral SI (areas 1, 3b; x = 46, y = −30, z = 60) and SII (OP 4; x = 48, y = −8, z = 10); see Figure 3A. BOLD time courses for these conditions are shown in Figure S1. Contrasting patterned with random stimulus trials revealed an increased activation in contralateral SI (areas 3b, 1, 2; x = 42, y = −30, z = 56) and anterior superior parietal cortex (aSPC; area 7; x = 30, y = −48, z = 54); see Figure 3B and Figure S2. Analysis of interaction effects revealed no significant results.

We further investigated BOLD signal changes for the two possible pattern orientations (upward and downward diagonals). Within the activations of moving vs. stationary stimulation, the analysis revealed directionality differences for moving patterns in contralateral SI (area 1; x = 38, y = −38, z = 64) and SII (OP 4; x = 52, y = −8, z = 8), as shown in Figure 3C. Within the activations of patterned vs. random stimuli, differential orientation effects for stationary patterns were found in right aIPS (area hIP3; x = 36, y = −48, z = 50); see Figure 3D. Closer inspection of these motion directionality and pattern orientation effects revealed that the upward diagonal motion direction showed by tendency increased activity in SI and SII compared with the downward diagonal motion direction. Likewise, the upward diagonal pattern orientation tended to be stronger represented in aIPS, compared with the downward diagonal pattern orientation. In terms of behavior (assessed outside the scanner), however, there were no significant differences between participants' performance in identifying upward and downward oriented patterns and motion directions.

**Figure 4:**
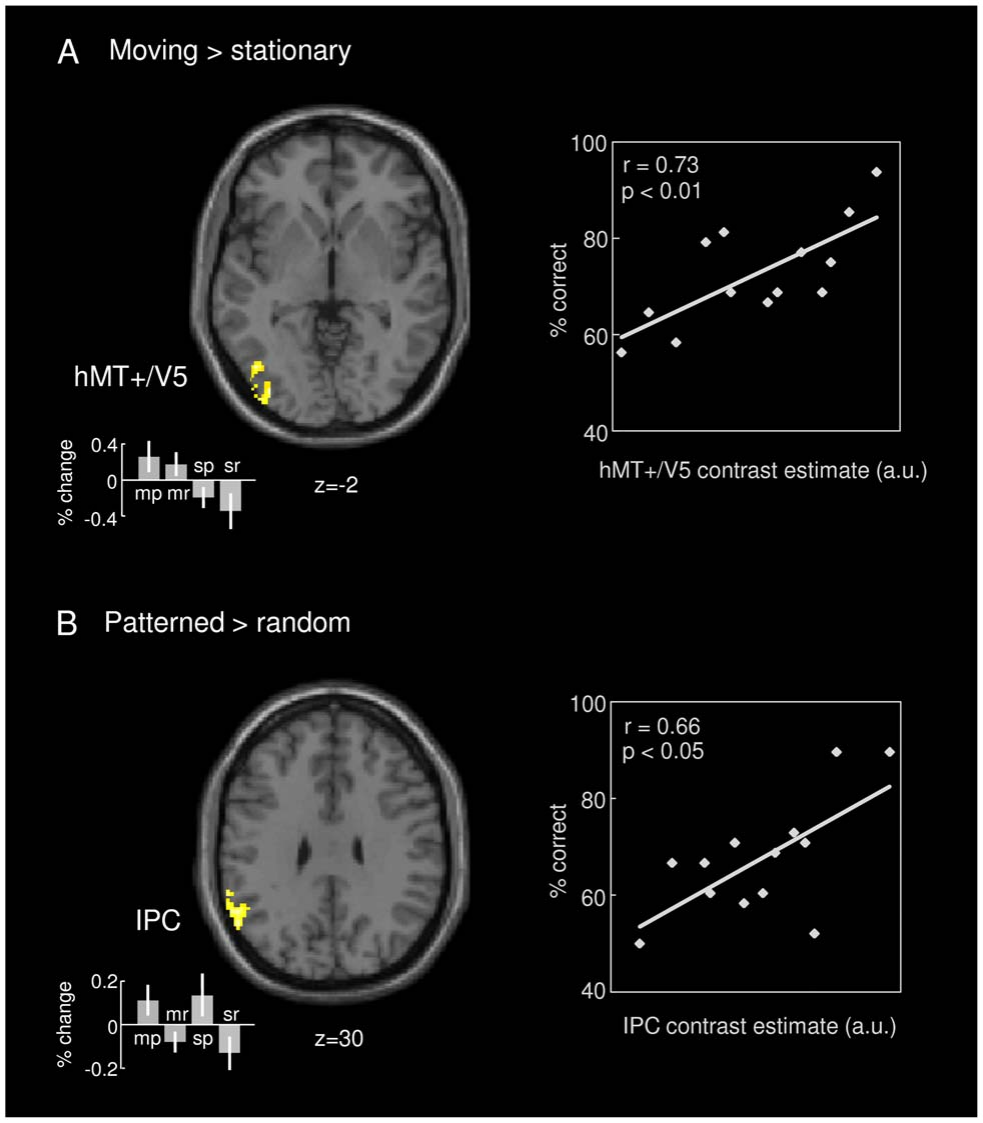
Areas involved in tactile motion and pattern processing. A. Contrasting moving with stationary trials revealed an increased BOLD response in medial superior parietal cortex (not visible) and middle temporal cortex (hMT+/V5; on the left). Individual subjects' contrast estimates in hMT+/V5 correlated positively with their accuracy in identifying moving stimuli correctly (on the right). B. Contrasting patterned with random stimulus trials revealed an increased BOLD response in inferior parietal cortex (IPC; on the left). Individual subjects' contrast estimates in IPC correlated positively with their accuracy in identifying patterned stimuli correctly (on the right). A-B. Effect sizes are plotted in terms of % signal change for all four stimulus types: moving patterned (mp), moving random (mr), stationary patterned (sp), and stationary random (sr). (Group-level analysis; p_cluster_<0.05, whole-brain FWE corr.).

Next, we tested differential effects of motion and pattern conditions in a whole-brain analysis, that is, also including areas that may not have been generally activated by tactile stimulation. The comparison between moving and stationary stimuli revealed increased activity in middle temporal cortex (area hMT+/V5; x = −44, y = −70, z = −2) and medial aSPC (area 5; x = −10, y = −40, z = 56) in the left hemisphere (Figure 4A, left, and Figure S3 for BOLD time courses). The hMT+/V5 activation remained also statistically significant after small volume correction with the anatomical ROI of hMT+/V5 defined using the Anatomy toolbox for SPM8. 15% of this ROI were activated during tactile motion processing. The overlap is shown in Figure S4. To assess to what extent these areas may have contributed to conscious processing of tactile motion, we investigated performance-dependent covariation in these areas. Interestingly, the BOLD responses in area hMT+/V5, but not medial aSPC, correlated positively with subjects' accuracy in identifying moving stimuli correctly (Figure 4A, right). Comparing patterned and random stimulus trials revealed increased activity in left inferior parietal cortex (IPC; x = −60, y = −56, z = 32), involving parts of the supramarginal and angular gyri; see Figure 4B (left) and Figure S3. Again, the BOLD responses in this area correlated positively with subjects' accuracy in identifying patterned stimuli correctly (Figure 4B, right). Control analyses showed no correlation between motion-related responses in hMT+/V5 and participants' accuracy in pattern identification and between pattern-related responses in IPC and their accuracy in motion identification (both r's<0.2, p's>0.5). In addition, there were no other areas significantly activated in all presented contrasts besides the reported ones.

**Figure 5:**
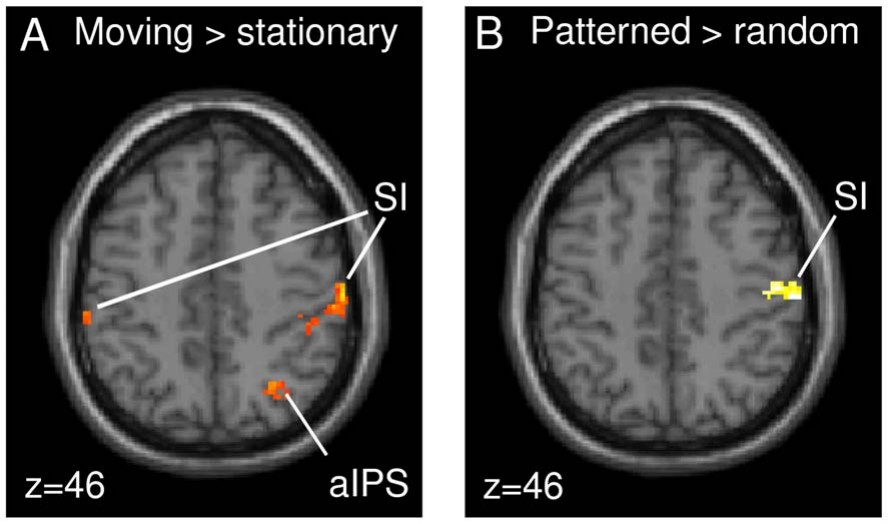
Psychophysiological interaction analyses using left hMT+/V5 and left IPC as seed regions. A. The interaction term for moving vs. stationary trials revealed a significant increase in coupling between left hMT+/V5, bilateral SI, and right anterior intraparietal sulcus (aIPS) during motion processing. B. For patterned vs. random stimulus trials, the coupling between left IPC and right SI was significantly increased. (Group-level analysis; p_cluster_<0.05, whole-brain FWE corr.).

On the basis of the GLM results above, we studied possible changes in effective connectivity of the areas that were identified as specifically related to tactile motion and pattern processing. To this end, PPI analyses were performed using the peak voxels (spheres of 5 mm) of the motion and pattern activations located in left hMT+/V5 and left IPC, respectively, as seed regions for exploring coupling to the rest of the brain (whole-brain analysis). The psychophysiological interaction term created for moving vs. stationary stimuli revealed a significant increase in coupling between left hMT+/V5, bilateral SI (ipsilateral: area 2; x = −48, y = −24, z = 42; contralateral: area 1; x = 56, y = −32, z = 52), and right aIPS (area hIP3; x = 30, y = −62, z = 46) during motion processing (Figure 5A). The PPI for patterned vs. random stimulation revealed a significant increase in effective connectivity between left IPC and right SI (areas 1, 2, 3b; x = 58, y = −22, z = 46) during pattern processing (Figure 5B).

## Discussion

The present study examined the neuronal networks underlying the processing of tactile motion and pattern, using high-field fMRI under tightly controlled passive stimulation conditions. Compared to matched control stimuli, stimulus-specific BOLD responses for both moving and patterned stimuli were already evident in early stages of somatosensory processing. Moreover, in line with previous work, tactile motion evoked activity in hMT+/V5, an area traditionally associated with visual motion perception. An analysis of effective connectivity further revealed that the responses in area hMT+/V5 were functionally coupled to a somatosensory network including primary somatosensory cortex (SI) but also anterior intraparietal sulcus (aIPS; area hIP3). The processing of tactile patterns, in contrast, was characterized by distinct responses in inferior parietal cortex (IPC), and this activity was found to be functionally coupled directly to the responses in SI. Furthermore, both hMT+/V5 and IPC showed a significant correlation between task-induced neuronal activity and individual performance in identifying the respective stimulus attribute.

Overall, tactile stimulation of the fingertip activated a widely distributed neuronal network including the contralateral SI (areas 3b, 1, 2) and bilateral secondary somatosensory cortex (SII; OP 1, 4), aIPS (areas hIP3, hIP2), inferior frontal gyrus (IFG; area 44), lateral prefrontal cortex (LPFC), as well as the pre-supplementary motor area (pre-SMA; area 6), insular cortex, thalamus, and the cerebellum, which altogether is in line with previously reported functional networks active during tactile processing (e.g., [24], [40], [44], [59], [60]).

Within the overall network associated with tactile stimulation, processing of tactile motion was reflected by increased activity in contralateral SI (areas 3b, 1) and SII (OP 4). Moreover, both SI (area 1) and SII (OP 4) were found to respond differentially to different motion directions. Selective encoding of tactile motion by populations of SI neurons has been observed in invasive recordings in monkeys [11]–[13]. More recently, Pei and colleagues [61] proposed that direction tuning in SI first emerges in area 3b, and is elaborated in area 1 to yield a more invariant representation of motion direction. In addition to SI, stimulus-specific firing of SII neurons during passive touch of moving gratings was shown in monkeys by Pruett and colleagues [62]. Whereas a general role of somatosensory cortex during tactile motion processing has also been reported in previous functional imaging work [21]–[23], the present evidence for differential BOLD signal changes for motion direction may not have been expected a priori. Thus far, direction- or orientation-selective responses in human sensory cortices have only been demonstrated using pattern classification methods for fMRI data analysis [63]–[65]. Because we did not investigate orientation encoding systematically in the present study and cannot fully exclude stimulus confounds, such as different skin indentation due to varying stimulus orientations, further studies are needed to explore the cause of the observed effect in more detail. However, the present evidence for motion-specific responses in human SI and SII complements the invasive findings and suggests that somatosensory cortex downstream from area 3b may similarly contribute to the emergence of an invariant representation of tactile motion in both types of species.

Replicating previous functional imaging work [29]–[31], the present analysis further revealed robust activation of area hMT+/V5 during processing of tactile motion. Area hMT+/V5 was long treated as a purely visual motion-sensitive area, but has recently been shown to be engaged during processing of motion in other sensory modalities as well [29], [30]. Interestingly, in Blake et al.’s work [30] a matched visual imagery condition did not activate hMT+/V5 significantly, which has been taken as evidence that the area's engagement during tactile motion cannot be fully explained by covert mental visualization of the somatosensory input. Involvement of area hMT+/V5 in tactile motion processing was consistently observed in both congenitally blind and sighted subjects [32]–[34]. Sani and colleagues further proposed a functional segregation of area hMT+/V5 in anterior and posterior subregions differentially involved in multisensory (visual and tactile) and visual motion processing [34], which might conform to areas MST and MT [44]. In line with this previous evidence, here, we found activation of hMT+/V5 extending towards anterior regions during processing of abstract, non-naturalistic Braille-like patterns, which may have been relatively difficult to imagine visually (cf. [43], [66]). Furthermore, tactile motion in the present experiment was manipulated within a subset of non-target stimuli, while the subjects' task consisted of detecting a markedly distinct target pattern, such that task demands did not encourage active visualization of the non-target's specific features. Notably, however, the hMT+/V5 responses evoked by moving stimuli covaried with subjects' ability to identify this type of stimuli outside the scanner, which supports the view that hMT+/V5 may in particular contribute to conscious perception of tactile motion.

PPI analysis of effective connectivity revealed that activity in area hMT+/V5 was not only increased but also functionally coupled to the responses in SI (areas 1, 2) and aIPS (area hIP3) during tactile motion processing. The ventral part of the aIPS has been shown to be engaged in multisensory motion processing [29], [31], [48], indicating that there might exist a human equivalent of the motion-sensitive area VIP within the monkey IPS. The present evidence for increased functional coupling of both primary somatosensory and motion-sensitive areas with hMT+/V5 may thus suggest transfer of somatosensory information to the motion-specialized area hMT+/V5 in visual cortex independent of the recruitment of other visual association areas such as precuneus [67], [68], which renders purely visual imagery as an explanation unlikely. Instead, the course of information processing might directly involve somatosensory cortex, hMT+/V5, and aIPS, given that neurons in monkey VIP receive projections from several visual areas (especially MT+/V5), and from motor, somatosensory, auditory, and other multisensory cortices [69]. These findings might indicate a more general role for hMT+/V5 in terms of multisensory motion processing (see also [34]). Similar conclusions have been drawn for visual and tactile object processing in the lateral occiptital complex (LOC; see [70], [71]).

In addition to the motion-specific responses in SI, SII, and in hMT+/V5, moving stimuli evoked increased activity in the medial part of the left anterior superior parietal cortex (aSPC; area 5). The superior parietal cortex is known to be involved in various cognitive processes, in particular somatosensory and sensorimotor integration as well as visuospatial attention and memory, whereas the aSPC was shown to integrate information mainly from the somatosensory cortex [72], [73]. In the present experimental context, medial aSPC activity neither covaried with subjects' behavioral performance nor reflected the stimuli's motion direction, and was not functionally coupled to the motion network outlined above, thus likely reflecting a less direct involvement in somatosensory information processing.

Like tactile motion, tactile patterns also evoked increased activity in SI (areas 3b, 1, 2), compared to randomly structured control stimuli, which is in agreement with previous work on tactile form perception in humans [23], [24] and monkeys [18]–[20]. Pattern processing furthermore engaged the aSPC (area 7), corroborating previous evidence that this part of the aSPC, which receives direct projections from area 2 in SI [72], [73], is critically involved in somatosensory processing of shape information [74]. Moreover, complementing electrophysiological evidence for the existence of orientation-tuned neurons in somatosensory areas [14]–[17], we found differential BOLD responses to different pattern orientations in the aIPS (area hIP3), which underpins this multisensory area's role in the processing of tactile object features (for related evidence, see, e.g., [23], [24], [26], [49], [50]). As with the differential effects for motion direction, the present finding of differences for pattern orientations using fMRI was rather unexpected. There is, however, recent evidence for coarse-scale orientation maps in V1 measured using fMRI [75], which indicates that selective population responses may in principle be assessed also with univariate statistical analysis. Although their functional significance has to be investigated in further studies, these findings support the existence of a putative human equivalent of the monkey AIP, which is known to be engaged in tactile and visual object processing [46].

In addition to the pattern-specific responses in SI, aSPC, and aIPS, which were also activated during tactile stimulation per se, the whole-brain analysis revealed that tactile pattern processing further recruited an area in the left inferior parietal cortex (IPC), including parts of the supramarginal and angular gyri. Associative somatosensory function was early attributed to the IPC in several lesion studies [27], [28], [76]. For instance, Reed and colleagues [28] reported a patient's impairment of shape recognition specific to the tactile modality resulting from a small, inferior parietal infarction. In an fMRI study by Deibert and colleagues [77], the inferior parietal lobule (supramarginal and angular gyri) was activated when subjects manipulated and identified an object's shape. More recently, Miquée and colleagues [78] investigated fMRI correlates of haptic shape perception in a task divided in shape-encoding and -matching steps, and showed that the IPC was specifically involved in shape matching. The latter finding supports the view of the IPC as a neuronal substrate of shape representation rather than coordination of finger movements as required in tactile object recognition tasks. The present results confirm this conclusion, demonstrating IPC activity during passive touch of patterned Braille-like stimuli. Furthermore, the BOLD responses in IPC covaried with participants' individual ability to identify patterned stimuli (assessed outside the scanner), providing evidence that the IPC may in particular contribute to conscious perception of tactile patterns. Finally, our PPI analysis showed that pattern processing entailed specific functional interactions between IPC and contralateral SI, which indicates that the IPC was intimately involved in the exchange of modality-specific somatosensory information. This exchange of tactile information might potentially occur by projecting from SI to IPC via SII, given the evidence for anatomical connections between SI and SII as well as between SII and IPC [79]. In line with this proposal, using a less stringent significance threshold of p<0.005 (uncorr.) for our PPI analysis, we found suggestive evidence for a role of SII as part of this specific functional network, which remains to be explored more fully in the future.

The engagement of IPC during tactile pattern processing was in many respects phenomenologically similar to the engagement of hMT+/V5 during tactile motion processing. Both areas were selectively recruited by the presence of an abstract tactile stimulus attribute (pattern or motion), were functionally coupled to somatosensory cortex in a stimulus-dependent manner, and the specific responses in both areas covaried with subjects' perceptual performance. With respect to the particular significance of these areas in the processing of tactile stimulus attributes, the present results may suggest that in analogy to the visual system, modality-specific somatosensory areas may interact with regions that are dedicated to the integration of specific perceptual features, such as motion or pattern, into a conscious perceptual concept. Such a concept might not necessarily be modality-specific and seems to involve not only designated somatosensory (IPC) or multisensory areas (aIPS) but, in the case of tactile motion, also area hMT+/V5.

In sum, our results corroborate that somesthesis of specific stimulus attributes engages characteristic processing networks that, on the one hand, involve modality-specific somatosensory areas, but, on the other hand, incorporate multisensory or even acknowledged visual areas. This overall picture is in line with increasing evidence that processing of sensory input from any specific modality may result in an abstract, essentially multisensory representation (e.g., [29], [31], [39], [44], [52], [70], [71], [80]). Thereby, areas specialized in the processing of abstract object attributes such as motion and pattern may be more process- than modality-driven. The present findings support this integrative view, and indicate that early, modality-specific representations of tactile information may be directly relayed to cortical areas that are dedicated to the further processing of specific stimulus features, regardless of their sensory modality.

## Supporting Information

Figure S1 **BOLD time courses for moving and stationary stimuli.** Group-averaged BOLD time courses, time-locked to stimulation onset, extracted from SI (A) and SII (B) for moving and stationary trials.
(PDF)

Figure S2 **BOLD time courses for patterned and random stimuli.** Group-averaged BOLD time courses, time-locked to stimulation onset, extracted from SI (A) and anterior superior parietal cortex (aSPC; B) for patterned and random stimuli.
(PDF)

Figure S3 **BOLD time courses for hMT+/V5 and IPC.** Group-averaged BOLD time courses, time-locked to stimulation onset, extracted from hMT+/V5 for moving and stationary trials (A) and from inferior parietal cortex (IPC) for patterned and random stimuli (B).
(PDF)

Figure S4 **Overlap with anatomically defined ROI for hMT+/V5 activation.** The part of the activation for moving vs. stationary stimuli that overlaps with the anatomically defined ROI for hMT+/V5 is shown in red and superimposed on the probabilistic map provided by the Anatomy toolbox for SPM.
(PDF)
