# Supplementary figures and images for: Tactile Motion and Pattern Processing Assessed with High-Field fMRI
Source: PLoS One. 2011 Sep 15;6(9):e24860. doi: 10.1371/journal.pone.0024860 (PMC3174219; doi:10.1371/journal.pone.0024860)

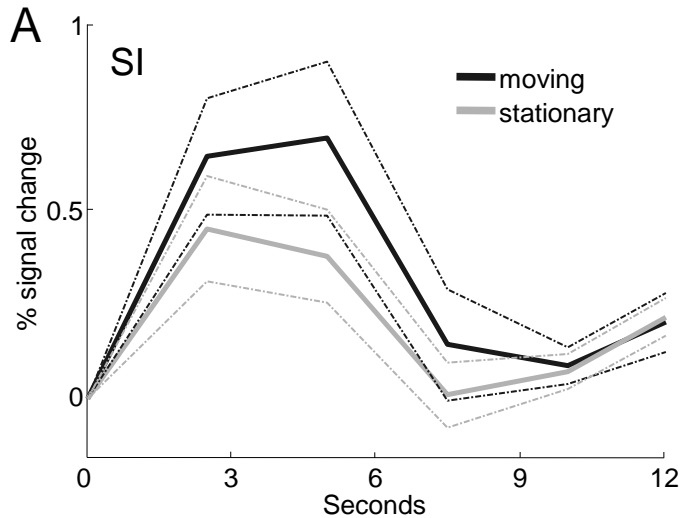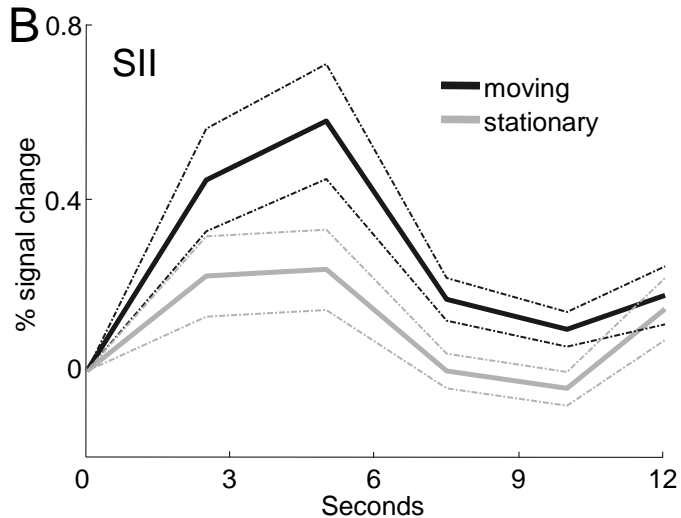

Supplement: Figure S1 — BOLD time courses for moving and stationary stimuli. Group-averaged BOLD time courses, time-locked to stimulation onset, extracted from SI (A) and SII (B) for moving and stationary trials. (PDF) [file pone.0024860.s001.pdf]

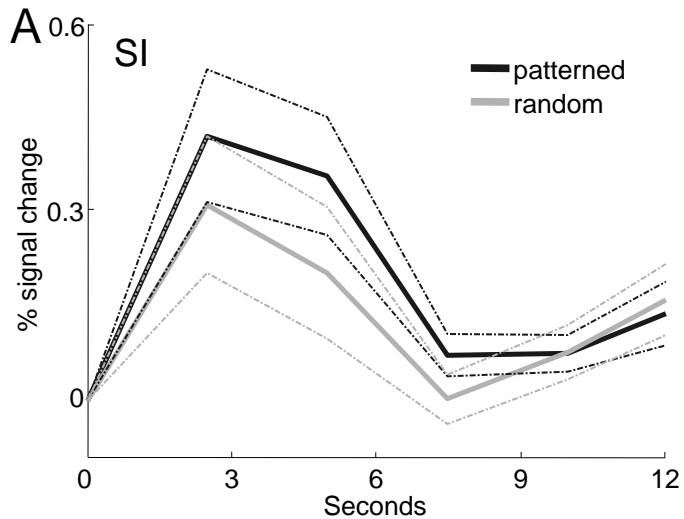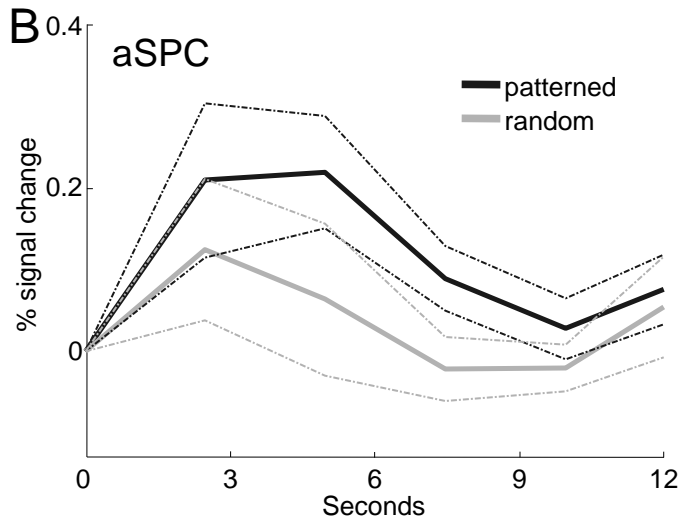

Supplement: Figure S2 — BOLD time courses for patterned and random stimuli. Group-averaged BOLD time courses, time-locked to stimulation onset, extracted from SI (A) and anterior superior parietal cortex (aSPC; B) for patterned and random stimuli. (PDF) [file pone.0024860.s002.pdf]

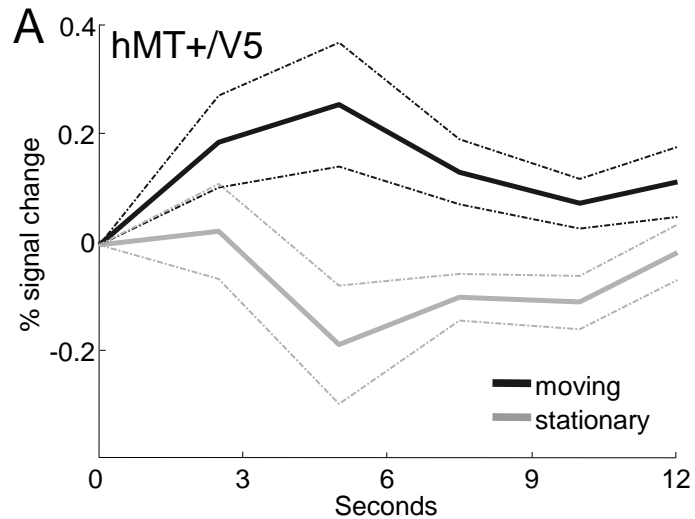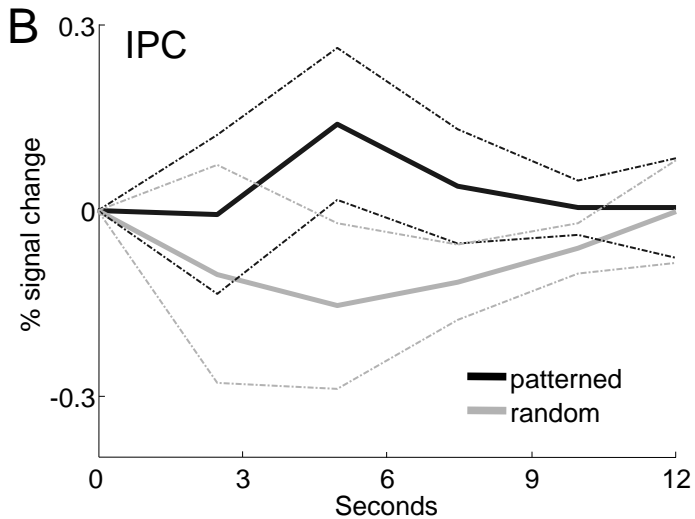

Supplement: Figure S3 — BOLD time courses for hMT+/V5 and IPC. Group-averaged BOLD time courses, time-locked to stimulation onset, extracted from hMT+/V5 for moving and stationary trials (A) and from inferior parietal cortex (IPC) for patterned and random stimuli (B). (PDF) [file pone.0024860.s003.pdf]

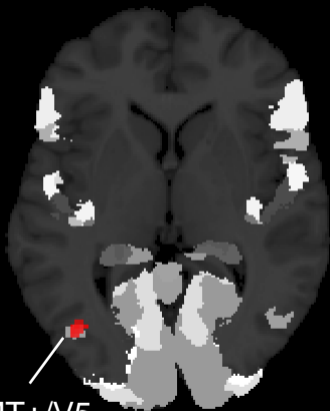

hMT+/V5

$z=-2$

Supplement: Figure S4 — Overlap with anatomically defined ROI for hMT+/V5 activation. The part of the activation for moving vs. stationary stimuli that overlaps with the anatomically defined ROI for hMT+/V5 is shown in red and superimposed on the probabilistic map provided by the Anatomy toolbox for SPM. (PDF) [file pone.0024860.s004.pdf]
